# Advances in Xylooligosaccharides Production From Lignocellulosic Materials and Its Application as Prebiotics

**DOI:** 10.1111/1750-3841.71379

**Published:** 2026-08-25

**Authors:** Paula Cavion Costa, Marli Camassola

**Affiliations:** ^1^ Enzymes and Biomasses Laboratory University of Caxias do Sul Caxias do Sul Rio Grande do Sul Brazil

## Abstract

With the growing interest in functional foods and their role in promoting health beyond basic nutrition, increasing attention has been directed toward the modulation of gut microbiota as a key determinant of human well‐being. In this context, prebiotics and probiotics have emerged as central bioactive ingredients due to their capacity to selectively influence microbial composition and metabolic activity in the gastrointestinal tract. Prebiotics are defined as nondigestible food components that are selectively utilized by beneficial microorganisms, leading to improved host health, whereas probiotics are live microorganisms that, when administered in adequate amounts, confer health benefits to the host. This review explores the mechanisms of action, major sources, and documented health effects of both prebiotics and probiotics, with particular emphasis on their roles in digestive health, immune modulation, and the prevention or management of metabolic and inflammatory disorders. Special attention is given to xylooligosaccharides (XOS) as emerging prebiotics derived from lignocellulosic biomass, highlighting recent advances in enzymatic production processes using microbial enzymes, especially fungal xylanases. Furthermore, the paper discusses current biotechnological strategies for sustainable production, purification, and formulation of prebiotic and probiotic ingredients, as well as the growing evidence supporting their synergistic interaction when combined as synbiotics. Understanding the complex interplay between prebiotics, probiotics, and the gut microbiota is essential for the rational design of next‐generation functional foods and dietary interventions aimed at improving human health.

**Abbreviations**
ABF: *α*‐*L*‐arabinofuranosidase
AGU: *α*‐glucuronidase
AXE: acetyl‐xylan esterase
AXOS: arabinoxylooligosaccharides
CAGR: compound annual growth rate
DP: degree of polymerization
EFSA: European Food Safety Authority
FAE: feruloyl esterase
FAO: Food and Agriculture Organization
FOS: fructooligosaccharides
GE: glucuronoyl esterase
GHs: glycoside–hydrolases
GOS: galactooligosaccharides
LCC: lignin–carbohydrate complex
NDOs: nondigestible oligosaccharides
NDS: nondigestible sugars
NGP: next‐generation probiotics
QPS: qualified presumption of safety
SCFA: short‐chain fatty acids
WHO: World Health Organization
XOS: xylooligosaccharides

## 1 Introduction

The concept of prebiotics has evolved over the years and has now reached a consensus. It is a substrate selectively utilized by the host‐associated microorganisms conferring a health benefit. It is considered a fermented ingredient, nondigestible sugar (NDS) that results in specific changes in the composition and/or activity of the gastrointestinal microbiota (Gibson et al. 2017). The increase in cases of gastrointestinal disturbances due to the changes in food consumption and people's lifestyle combined with the demand for functional ingredients and products contributes to the prebiotics’ demand for use (Ahuja 2021; Valladares‐Diestra et al. 2023). Functional foods are items that confer more health benefits than the elemental, enhanced or with the addition of ingredients that are biologically active (Victoria Obayomi et al. 2024).

Recent studies show that the prebiotics market has surpassed the mark of $4.95 billion in 2020 and is expected to grow 10.2% between 2021 and 2027. Prebiotics stimulate beneficial microorganisms in the human gut and can be used as sweeteners and diet low‐calorie food (Brienzo et al. 2010; Victoria Obayomi et al. 2024). Xylooligosaccharides (XOS) are ingredients with probiotic properties and can, therefore, be produced and commercialized as functional food. They increase calcium absorption, confer protection against cardiovascular diseases, increase nutrient absorption, inhibit pathogenic microorganisms, reduce the risk of colon cancer, promote an immunological response, have antioxidant capacity, as well as antiallergic and anti‐inflammatory effects. It is known that prebiotics promote inhibition of pathogens and stimulate the immune system, reduce blood lipid levels, alter insulin resistance, on metabolites that influence brain function, energy, and cognition, as well as mineral bioavailability (Farias et al. 2019; Gibson et al. 2017; Poletto et al. 2020). The main bottleneck for XOS dissemination is the cost of this prebiotic type in comparison to others, such as fructooligosaccharides (FOS), galactooligosaccharides (GOS), among others (Jain et al. 2015; Mano et al. 2018; Valladares‐Diestra et al. 2023).

Probiotics, live microorganisms that in adequate amounts confer health benefits for the host, can restore the balance of the intestinal ecosystem when in imbalance (dysbiosis) (Martín and Langella 2019). Prebiotics, in a symbiotic association with probiotic food and intestinal flora, promotes the growth of beneficial bacteria and its survival, showing a positive effect in the large intestine (Gibson et al. 2017; Pinales‐Márquez et al. 2021). The combination of these two products is called symbiotics and can be defined as a mixture that beneficially affect the host by improving the survival and implantation of live microbial dietary supplements in the gastrointestinal tract, by selectively stimulating the growth and/or by activating the metabolism of one or a limited number of health‐promoting bacteria, thus improving the host welfare (Gibson et al. 2004).

Accordingly, this review distinguishes itself by critically integrating recent advances in the enzymatic bioconversion of lignocellulosic biomass (LCB), precision strategies for controlling the structural features of XOS—including degree of polymerization (DP) and substitution patterns—and functional evidence from in vitro systems, animal models, and human clinical studies. By explicitly linking sustainable bioprocess development with mechanistic insights and clinically relevant metabolic outcomes, this work provides a previously underdeveloped translational framework that connects fundamental research with industrial implementation and health‐related applications. Moreover, the review identifies key knowledge gaps, methodological constraints, and regulatory barriers, thereby informing future interdisciplinary efforts aimed at enabling scalable, reproducible, and scientifically validated XOS‐based interventions.

## 2 LCB Structure

**FIGURE 1:**
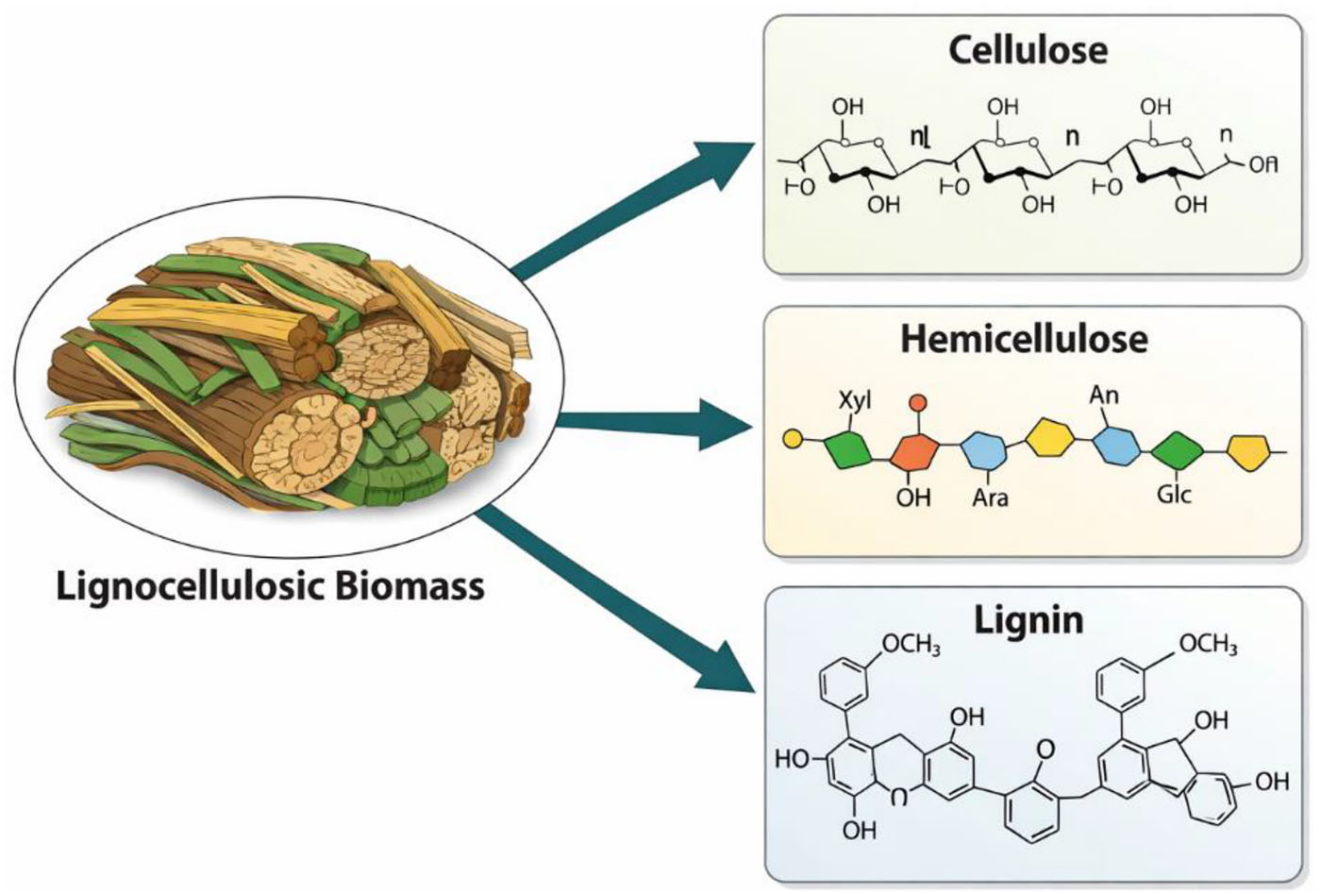
Lignocellulosic biomass major components and its structure.

LCB is the most abundant renewable carbon source in the world and is a residue of agroindustries that has great potential in conversion to fermentable sugars. Polysaccharides, the major constituents of vegetal biomass, can serve as a food reserve or to mechanical strength. Lignocellulosic is the farthest content found in plants and is a heterogeneous polymer composed mostly of cellulose, hemicellulose, and lignin. Their percentage can vary according to the biomass source (Fan et al. 2024; Gupta et al. 2016; Linares‐Pasten et al. 2016). Figure 1 shows the major components of LCB and its structure.

Cellulose, a high molecular weight molecule, is a homopolymer constituted by 1,4‐*D*‐glucopyranose units, with cellobiose units (two glucose molecules) linked by covalent β‐1,4 bonds. The DP depends on the source of the LCB. The linkages of this polymer allow its linear conformation. Hydrogen bonds are present between cellulose chains, which make them get an ordered form, known as the crystalline structure, whereas the structures without a good order are known as the amorphous part. Therefore, even being a homopolymer its structure is heterogeneous and rigid (Harmsen et al. 2010; Sajith et al. 2016).

Hemicellulose is the second most abundant polysaccharide fraction, accounting for approximately 20%–35% of LCB, whereas lignin generally contributes 10%–30% depending on the feedstock (Ebringerová and Heinze 2000; Peng et al. 2012). It is located between the macro and microfibrils of cellulose, as well as the lignin polymers. It is a heteropolymer composed of sugar acids (called uronic acids), hexoses (such as glucose, galactose, rhamnose, and mannose), and pentoses (arabinose and xylose). These structures are linked by β‐1,3 and β‐1,4 bonds. The percentage of monomeric units can vary among the biomass, and their name is given according to the most abundant unit. Due to the highly branched structure and the acetyl groups linked to the main chain, this polymer is not crystalline. Among the various hemicellulosic polysaccharides, xylan is the predominant fraction in most agricultural residues and hardwoods, representing up to 80%–90% of total hemicellulose in some biomass sources (Carvalho et al. 2013; Ebringerová and Heinze 2000). Xylan is composed of *β*‐*D*‐xylopyranose units linked by β‐1,4 bonds. It is insoluble in water under low temperatures, but its hydrolysis starts earlier than cellulose (Beg et al. 2001; Linares‐Pasten et al. 2016; Sajith et al. 2016).

In addition to their abundance in lignocellulosic feedstocks, xylans from different biomass sources have attracted increasing interest due to the biological activities associated with the resulting XOS (Table 1). Depending on their structural characteristics, including branching degree and substitution pattern, XOS may exert distinct physiological effects, such as selective stimulation of *Bifidobacterium* and *Lactobacillus* populations, enhancement of short‐chain fatty acid (SCFA) production, modulation of intestinal inflammation, and potential alleviation of gastrointestinal disorders, including irritable bowel syndrome (IBS) (Broekaert et al. 2011; Santibáñez et al. 2021). These findings reinforce the importance of exploring diverse LCB as sustainable feedstocks for the production of high‐value prebiotic ingredients.

**TABLE 1:** Xylan content, predominant hemicellulose type, and reported applications of XOS produced from different lignocellulosic biomasses.

Biomass source	Xylan content (%)	Predominant hemicellulose	Reported XOS applications and health benefits	References
Rice husk	18–25	Arabinoxylan	Improved gut microbiota modulation and potential alleviation of irritable bowel syndrome (IBS) symptoms through increased SCFA production	(Broekaert et al. 2011; Santibáñez et al. 2021)
Wheat straw	20–30	Arabinoxylan	Bifidogenic effect and stimulation of beneficial intestinal bacteria	(Samanta et al. 2015)
Corn stover (corn straw)	23–30	Glucuronoarabinoxylan	Production of prebiotic XOS with selective stimulation of Bifidobacterium spp.	(Carvalho et al. 2013)
Corn cob	30–35	Xylan	High XOS yields and enhanced SCFA production	(Akpinar et al. 2009)
Sugarcane bagasse	22–36	Arabinoxylan	Efficient substrate for XOS production; promotion of beneficial gut microbiota and antioxidant effects	(Bragatto et al. 2013; Santibáñez et al. 2021)
Elephant grass	24–31	Arabinoxylan	Promising source for XOS production due to high hemicellulose content	(Iyyappan et al. 2023)
Oat hulls	25–35	Arabinoxylan	Improvement of intestinal health and modulation of inflammatory responses	(Broekaert et al. 2011)
Hardwoods (eucalyptus, birch)	20–35	Glucuronoxylan	Source of XOS with prebiotic activity and potential use in functional foods	(Moure et al. 2006)
Barley husk	20–28	Arabinoxylan	Increased production of butyrate and improvement of colon health	(Samanta et al. 2015)

The structural diversity of xylan has significant implications for its industrial utilization. Beyond serving as a substrate for the production of XOS, xylan has been explored for the manufacture of biofuels, xylitol, furfural, biodegradable films, hydrogels, packaging materials, pharmaceutical excipients, and specialty chemicals (Mäki‐Arvela et al. 2010; Samanta et al. 2015). Nevertheless, the conversion of xylan into XOS remains one of the most attractive valorization routes because XOS are recognized as emerging prebiotics capable of selectively stimulating beneficial gut microorganisms while exhibiting high stability during gastrointestinal transit (Aachary and Prapulla 2011; Santibáñez et al. 2021).

Lignin is an amorphous heteropolymer composed of three units of phenylpropane (hydroxycinnamic acids): *p*‐coumaryl, coniferyl, and sinapyl alcohol. They are linked in a three‐dimensional structure, which gives recalcitrance to the plant cell wall. Besides the physical role of lignin, it is also important for preventing plant deterioration by insect attack and water transportation through the plant. According to the methoxy degree of the aromatic ring, the basic unit is *p*‐hydroxyphenyl (known as H lignin, non metoxylled, derived from the *p*‐coumaryl alcohol), guayacyl (known as G lignin, with one methoxy group, derived from the coniferyl alcohol), or syringyl (known as S lignin, with two methoxy groups, derived from the sinapyl alcohol). Lignin of softwoods contains more than 90% of G lignin, whereas hardwoods present more S lignin (Becker and Wittmann 2019; Harmsen et al. 2010; Sajith et al. 2016). Cellulose and hemicellulose are linked by hydrogen bonds, and lignin is linked covalently to hemicellulose through the lignin–carbohydrate complex (LCC). These bonds can be phenyl glucosides, benzyl ethers, y‐esters esters, ferulate esters, and hemiacetals (Tarasov et al. 2018).

In a lower quantity is pectin, a polymer usually composed of galacturonic methyl esterified acids liked by α‐1,4 bonds. It has four main structures: homogalacturonan, rhamnogalacturonan I, xylogalacturonan, and rhamnogalacturonan II. Pectin has an important role in the porosity and ionic control of the cell wall, as well as in subjacent cell adhesion, defense, and growth (Canteri et al. 2012).

## 3 Xylan‐Degrading Enzymes

Xylanolytic enzymes degrade xylans into fermentable monosaccharides, which can be applied in many industries, such as biofuels, food, animal food, pulp, and paper, among others. They can be produced by many microorganisms, such as fungi, bacteria, and actinomycetes, of which the filamentous fungi are the most popular (Li et al. 2022; Linares‐Pasten et al. 2016; Sharma et al. 2022).

Enzymes involved in xylan degradation can be divided into endo‐acting or exo‐acting enzymes. The first ones hydrolyze bonds in the interior of the xylan polymer chain, while the second ones hydrolyze xylan either in the reducing or nonreducing ends of the chain. They act on β‐1,3 or β‐1,4 linkages. The xylan backbone is primarily hydrolyzed by enzymes such as endo ‐*β*‐*D*‐xylanase and *β*‐*D*‐xylobiohydrolase, which belong to the glycoside hydrolase (GH) family and catalyze the cleavage of glycosidic linkages within the xylan main chain. *β*‐*D*‐xylosidase degrades the β‐1,4 linkages, preferably in oligosaccharides (Li et al. 2022; Zabel and Morrell 2020).

**FIGURE 2:**
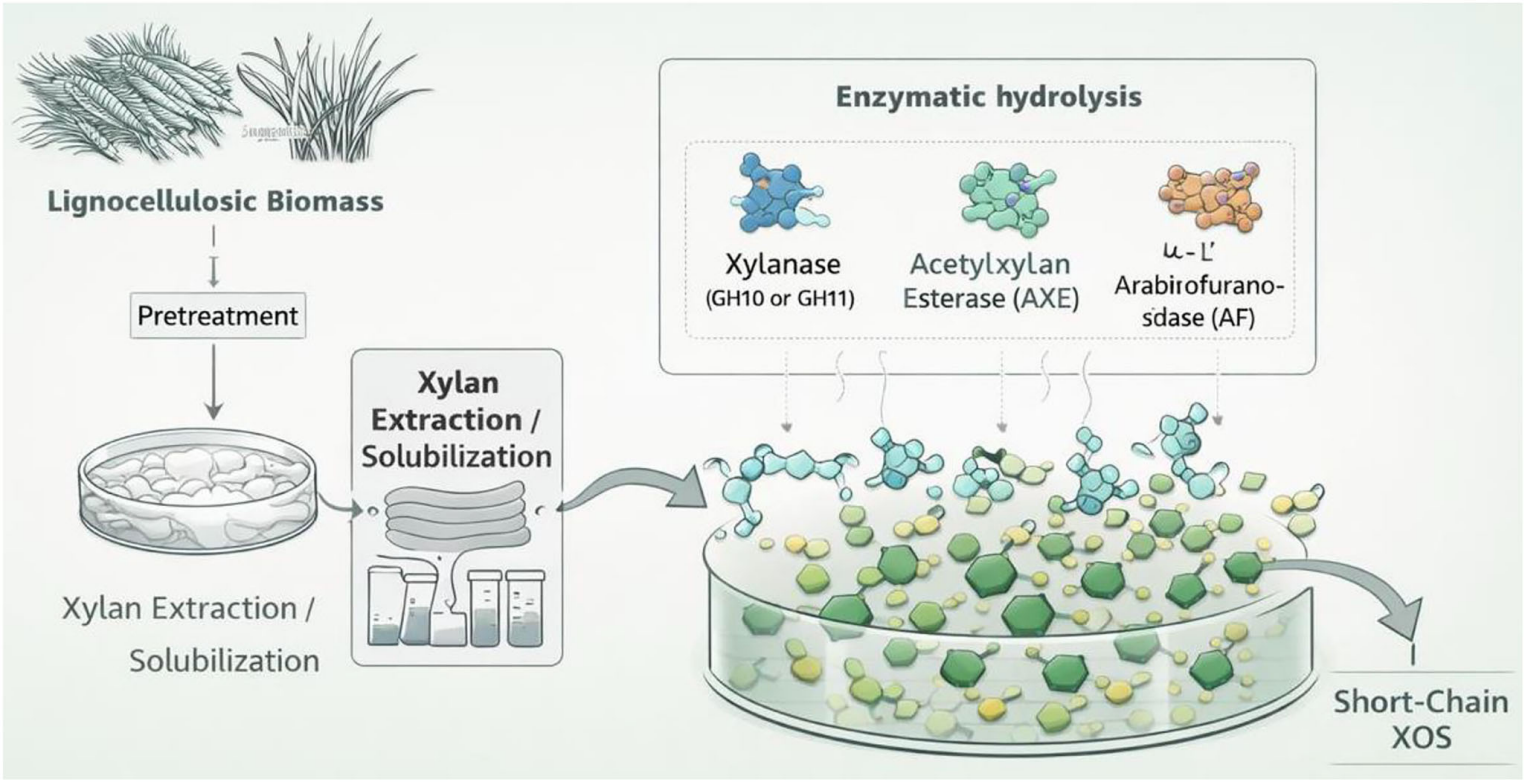
Enzymes acting on the main chain of xylose.

Xylan side‐chain enzymes are capable of removing the branches and side groups in different xylan types and include *α*‐*L*‐arabinofuranosidase (ABF), *α*‐glucuronidase (AGU), acetyl‐xylan esterase (AXE), feruloyl esterase (FAE), and glucuronoyl esterase (GE). ABF acts on the linkages between the residues of arabinose and xylosyl units, releasing arabinose. They are considered exo‐acting enzymes of the GH family and can be divided into Group A (remove arabinosyl residues at the position O‐2 or O‐3 in monosubstituted residues of xylose) and Group B (selectively release O‐3 arabinosyl from xylosyl residues with double arabinose). AGU also belongs to the GH family and hydrolyses the α‐1,2‐glycosidic linkages between xylosyl residues and (Me)GlcA. AXE hydrolyzes the ester linkages between the xylan backbone and acetyl groups, releasing acetic acid. FAE hydrolyzes the ester linkages between hydroxycinnamic acids and arabinosyl residues in the xylan. GE hydrolyzes the ester bonds between the (Me)GlcA side chain of glucuronoxylan and the aliphatic alcohols in lignin. Table 1 shows the enzymes that act on xylose and Figure 2 represents this action (Li et al. 2022; Zabel and Morrell 2020).

The study conducted by Morgan et al. (2017) showed that xylanase enzymes from GH 10 are more effective at producing short chain arabinoxylooligosaccharides (AXOS) from wheat arabinoxylans than family 11 enzymes or combinations of both. Specifically, the family 10 enzyme has broader substrate preference and lower specificity, favoring their action on substituted xylan chains and cleaving different sites along the backbone, favoring the formation of shorter oligomers, such as xylobiose and xylotriose. Conversely, GH11 enzymes prefer internal bonds and longer chains, often yielding different product profiles. The effects of temperature and exposure time on XOS production were more variable and depended on interactions with the specific xylanase family and pH conditions (Biely et al. 2016; Morgan et al. 2017; Scheller and Ulvskov 2010). This enzymatic diversity enables a more effective and selective depolymerization of the complex and heterogeneous hemicellulosic matrix when compared to systems relying on a limited number of catalytic activities.

From a bioconversion perspective, fungal xylanases are particularly attractive because many of them exhibit optimal activity under acidic pH and moderate to high temperatures, conditions that are compatible with industrial biomass processing and reduce the risk of microbial contamination (Polizeli et al. 2005; Vaishnav et al. 2018). Moreover, the controlled enzymatic hydrolysis of pretreated LCB using fungal xylanases allows the preferential release of XOS rather than complete depolymerization to xylose, which is a critical requirement for the production of prebiotic compounds (Mobarec et al. 2021).

XOS generated through fungal enzymatic systems have been extensively reported to selectively stimulate the growth of beneficial gut microorganisms such as *Bifidobacterium* and *Lactobacillus*, while remaining resistant to digestion in the upper gastrointestinal tract (Aachary and Prapulla 2011; Amorim et al. 2019). In this context, the use of fungal‐derived hemicellulases represents a strategic advantage, as these enzymes can be tailored through fermentation optimization or enzyme selection to modulate the DP and substitution pattern of XOS, factors known to influence their prebiotic efficacy (Finegold et al. 2014; Valladares‐Diestra et al. 2022; Valladares‐Diestra et al. 2023). Overall, recent studies reinforce that fungi‐based enzymatic platforms offer a sustainable and efficient route for the valorization of LCB into high‐value functional ingredients, aligning biomass bioconversion with circular bioeconomy principles and expanding the industrial applicability of hemicellulose‐derived products (Bhatia et al. 2020; Kumar and Chandra 2020). Table 2 presents data on hemicellulose enzymes production for hemicellulose degradation from LCB.

**TABLE 2:** Enzymes acting on xylose.

Enzyme	Action	Substrate	Product	Family
Endo‐*β*‐1,4‐xylanase	Acts on the inside xylan main chain and hydrolysate them into xylan oligomers and a small quantity of free xylose	Xylan polymer	Xylose and xylooligosaccharides	EC 3.2.1.8
*β*‐1,4‐xylosidase	Hydrolysates the xylooligosaccharides produced by endo‐1,4‐*β*‐xylanase into individual xylose units	Xylooligosaccharides, xylobiose and xylan fragments	Xylose	EC 3.2.1.37
*α*‐Glucuronidase	Hydrolyzes α‐1,2 bonds between glucuronic acid and xylose, separating the side chains of 4‐*O*‐metylglucuran	Side chains of 4‐*O*‐metylglucuran	Glucuronic acid	EC 3.2.1.139
*α*‐*L*‐arabinofuranosidase	Removes *L*‐arabinose side chains that are attached to the xylan backbone, increasing the effect of endo‐xylanases	*α*‐*L*‐arabinofuranosyl residues	*L*‐arabinose	EC 3.2.1.55
Acetylxylan esterase	Removes acetyl substituent groups from xylose, which can be inhibitory to the action of other enzymes	Acetyl substituents in xylose	Acetyl groups	EC 3.2.1.72
Ferulic acid esterase	Removes the ferulic acid from arabinofuranose sugar	Arabinofuranose	Ferulic acid	EC 3.2.1.73
*p*‐coumaric acid esterase	Breaks down the ester bonds between *p*‐coumaric acid and arabinose residues in xylan	Hemicellulose–lignin complex	*p*‐coumaric acid	

Although filamentous fungi remain the primary industrial producers of xylanases due to their high secretion capacity and broad spectrum of hemicellulolytic enzymes, increasing attention has been directed toward probiotic bacteria and yeasts as alternative sources of xylanolytic enzymes. Several species of lactic acid bacteria, including *Lactobacillus plantarum*, *Lacticaseibacillus rhamnosus*, *Lactobacillus brevis*, and *Bifidobacterium* spp., have demonstrated the ability to produce xylan‐degrading enzymes and utilize XOS as carbon sources, contributing to their prebiotic effects in the gastrointestinal tract (Broekaert et al. 2011; Patel and Goyal 2012). Similarly, probiotic and nonconventional yeasts, such as *Saccharomyces boulardii* and *Kluyveromyces marxianus*, have been investigated for their xylanolytic potential and application in lignocellulosic bioprocesses (Santibáñez et al. 2021). The integration of xylanase‐producing microorganisms with XOS production systems represents a promising strategy for developing synbiotic formulations, in which the generated XOS selectively stimulate the growth of beneficial microorganisms while simultaneously contributing to biomass valorization. Therefore, the exploration of bacterial and yeast‐derived xylanases may complement traditional fungal systems and expand the range of biotechnological applications associated with lignocellulosic biorefineries.

## 4 XOS: Structure and Benefits

XOS are oligosaccharides composed of five‐carbon sugars from xylose residues that vary in the DP from two to 10 molecules of xylan linked by β‐1,4‐xylosidic bonds, even though there are some authors in literature that consider their DP from two to 20 (Carvalho et al. 2013; Poletto et al. 2020). They have a wide variety of side groups, such as *⍺*‐*d*‐glucopyranosyl uronic acid, acetyl, 4‐*O*‐methyl‐*D*‐glucuronosyl, *L*‐arabinosyl, which is implied in its possible branched structure. The DP of XOS, as well as its side chains and linkages, can vary according to the source of xylan (Amorim et al. 2019).

The physiological functionality of XOS is strongly influenced by their DP. Although xylose and low‐DP oligosaccharides (DP2–DP4) are rapidly utilized by beneficial intestinal microorganisms, several studies have demonstrated that XOS with DP3–DP6 exhibit superior prebiotic properties due to their selective fermentation by *Bifidobacterium* and *Lactobacillus* species, resistance to digestion in the upper gastrointestinal tract, and prolonged availability in the colon (Aachary and Prapulla 2011; Broekaert et al. 2011; Moura et al. 2007). These oligomers have been associated with increased production of SCFAs, improved intestinal barrier function, modulation of gut microbiota composition, and reduced risks of gastrointestinal disorders and colorectal cancer (Samanta et al. 2015; Santibáñez et al. 2021). Consequently, controlling enzymatic hydrolysis conditions to maximize the production of XOS within the DP3–DP6 range is considered a key strategy for enhancing the functional value of lignocellulosic biorefineries.

These oligosaccharides are naturally found in honey, fruits, and vegetables, but not in enough amounts to have prebiotic effects. The use of lignocellulose biomass as a raw material to produce XOS is gaining attention in the scientific community, as it can be obtained from the most abundant component in the biome after cellulose. It is a valuable byproduct of agroindustry materials, also promising because of the valorization of biorefineries, and is gaining attention from the food and pharmaceutical industries due to their beneficial health properties. The market demand for XOS was expanding at a 2.7% compound annual growth rate (CAGR) between 2018 and 2022 and is expected to grow at a CAGR of 7% from 2023 to 2033 (FMI 2023).

XOS exert a range of beneficial effects on host physiology that stem primarily from their selective fermentation by gut microbiota. When XOS reach the colon intact, they are preferentially utilized by beneficial bacteria (especially *Bifidobacterium* spp. and, in some human studies, *Lactobacillus* spp.) leading to modulation of microbial composition and enhanced proliferation of health‐associated taxa. For example, supplementation of XOS in healthy adults promoted increases in fecal *Bifidobacterium* and *Lactobacillus* populations with good gastrointestinal tolerance at relatively low doses (1.4–2.8 g/day) compared to other prebiotics (Finegold et al. 2014; Lin et al. 2016; Linares‐Pasten et al. 2016).

Importantly, XOS fermentation results in the production of SCFAs, including acetate, propionate, and butyrate, which serve as energy substrates for colonocytes, reinforce mucosal barrier integrity, and help maintain immune homeostasis. SCFAs also reduce luminal pH, creating a gut environment that is less hospitable to pathogenic bacteria and more supportive of beneficial commensals (Nogueira‐Prieto et al. 2025).

Beyond microbial modulation and SCFA generation, XOS have demonstrated anti‐inflammatory and immunomodulatory effects in animal models. For instance, XOS supplementation in high‐fat diet–induced obese rats increased SCFA‐producing bacterial genera and attenuated colonic inflammation, as evidenced by reduced pro‐inflammatory markers and enhanced epithelial tight‐junction gene expression (Fei et al. 2020). Additional mechanistic work in preclinical immunosuppression models suggests that XOS may restore immune parameters, normalizing leukocyte counts and immunoglobulin levels after chemotherapeutic insult (Keung et al. 2025).

Clinical data further support XOS benefits for gut microbiota balance and gastrointestinal function. In a 6‐week dietary intervention, daily ingestion of rice porridge enriched with XOS significantly increased fecal counts of *Lactobacillus* and *Bifidobacterium*, while decreasing the abundance of *Clostridium perfringens* compared to placebo, illustrating improved microbiota balance in human subjects (Lin and Zhang 2017). Animal studies also demonstrate that XOS can alleviate constipation‐like symptoms and enhance SCFA production, supporting functional gut outcomes that are relevant to human gastrointestinal health (Song et al. 2023).

Emerging evidence suggests metabolic benefits as well. In rodent models of metabolic disturbance (e.g., high‐fat diet), XOS supplementation was associated with improved lipid profiles, reduced visceral adiposity, enhanced expression of genes involved in fatty acid oxidation, and strengthened intestinal barrier markers, which collectively suggest a role in metabolic regulation via gut–liver axis interactions (Li et al. 2021).

Collectively, these findings indicate that XOS confer multiple health benefits by shaping gut microbial communities, boosting SCFA production, enhancing gut barrier integrity, and modulating immune function. These effects, which can be observed in both human and animal studies, underscore the therapeutic potential of XOS as a prebiotic ingredient in functional foods and nutritional interventions (Nogueira‐Prieto et al. 2025).

## 5 XOS Production From LCB

From a technological standpoint, XOS can be generated by (i) direct extraction or partial depolymerization during physicochemical pretreatment of LCB, (ii) acid/chemical hydrolysis, or (iii) enzymatic hydrolysis of soluble or pretreated xylans. Chemical hydrolysis can be fast and high‐yielding but often lacks selectivity, produces high amounts of monomeric xylose, and requires neutralization and downstream purification steps; such conditions may also remove or alter biologically relevant substitutions. By contrast, enzymatic hydrolysis using endo‐1,4‐*β*‐xylanases and accessory debranching enzymes offers superior control over DP, milder reaction conditions, and higher selectivity for oligomer formation, enabling production of short XOS fractions with relatively low xylose content and preserved substitution patterns. These attributes make enzymatic routes the preferred choice for producing functionally consistent XOS for food and nutraceutical applications (Jain et al. 2015; Kumari et al. 2024; Liu et al. 2024; Palaniappan et al. 2021; Santibáñez et al. 2021).

Agro‐industrial residues (wheat straw, corn stover, sugarcane bagasse, sawdust, and spent mushroom substrates) have been effectively valorized into XOS using combinations of mild pretreatments (hydrothermal, microwave‐assisted, or alkaline solubilization) followed by enzymatic hydrolysis; these integrated approaches improve xylan accessibility and can increase short‐chain XOS yields while minimizing chemical usage. Process intensification (e.g., coupled hydrothermal pretreatment plus endo‐xylanase) and downstream fractionation techniques (membrane ultrafiltration, chromatography) are increasingly employed to obtain XOS fractions with target DP distributions and low monosaccharide content suitable for direct food formulation (Fareed et al. 2025; Kumari and Singh 2018).

A key benefit of enzymatic hydrolysis lies in the selectivity of the enzymes employed. Therefore, enzyme selection is a critical determinant of product profile. Appropriately chosen xylanolytic enzymes act preferentially on the xylan backbone, allowing the preservation of side‐chain substitutions on XOS molecules. This characteristic is particularly important, as the biological functionality and mode of action of XOS are influenced not only by their DP but also by their substitution pattern, which affects microbial selectivity and fermentation behavior in the gut (Valladares‐Diestra et al. 2023).

GH family 10 (GH10) and family 11 (GH11) xylanases differ in substrate specificity and cleavage patterns: GH10 enzymes typically display broader substrate tolerance and are efficient at generating short XOS (e.g., X2–X4) from substituted xylans, whereas GH11 enzymes can be more specific and may yield longer oligomers under certain conditions. Process variables—particularly pH, temperature, and reaction time—interact with enzyme family to shape yields and DP distribution; for instance, some studies report enhanced formation of small AXOS under acidic conditions when GH10 enzymes are used. These mechanistic insights justify the frequent use of tailored enzyme cocktails (combining endo‐xylanases with accessory ABFs, acetylxylan esterases, etc.) to maximize functional XOS output while limiting undesirable xylose release (Morgan et al. 2017; Rahmani et al. 2019).

**FIGURE 3:**
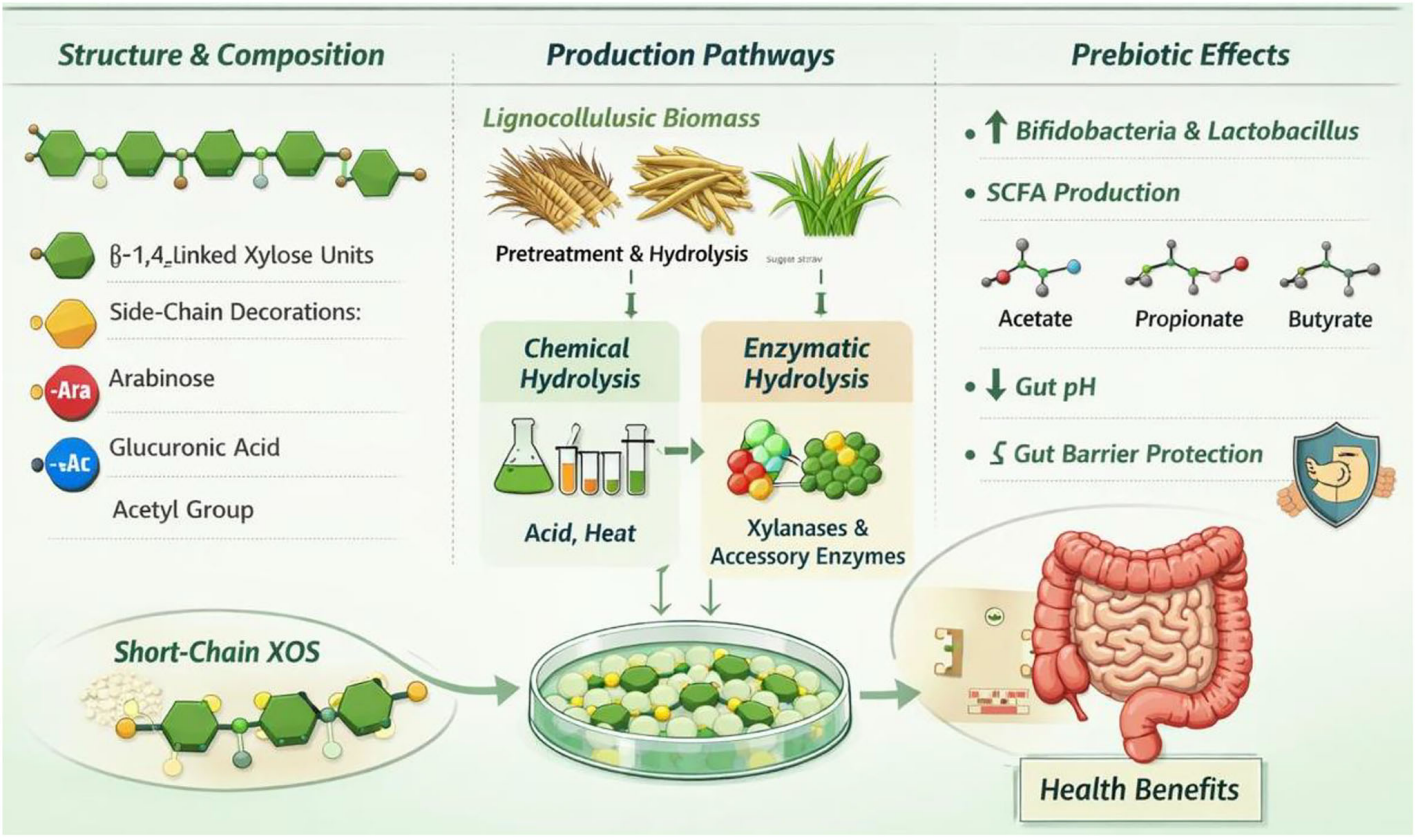
Xylooligossacharides structure, production pathways, and its prebiotic effects in humans.

When compared to the method using acid or alkaline hydrolysis, enzymatic production is relatively more economical, faster, sustainable, does not require special expensive equipment, and does not generate undesirable byproducts. Some bottlenecks involved in the production of XOS are a need for a low DP (X2 to X6) and a high degree of purity. Besides, heterogeneity in raw‐material composition and xylan structure across feedstocks complicates process standardization. Scale‐up requires balancing enzyme cost, reaction time, and downstream purification expenses to keep XOS economically competitive compared with other prebiotics. Finally, translating structural control into predictable in vivo functionality calls for systematic linking of specific DP/substitution profiles to microbial and metabolic endpoints in well‐designed human trials (Chen et al. 2021; Liu et al. 2024; Valladares‐Diestra et al. 2023). Figure 3 shows the steps for the production of XOS from LCB through different pathways and its benefits as prebiotics.

In this context, recent advances in enzymatic bioconversion have focused on the use of fungal endo‐xylanases to achieve controlled depolymerization of hemicellulosic substrates, enabling the preferential accumulation of low‐degree polymerization XOS while minimizing monosaccharide release (Wang et al. 2018). As summarized in Table 3, enzymes derived from *Penicillium*, *Aspergillus*, and thermophilic or recombinant fungal systems consistently demonstrate high selectivity toward X2–X3 fractions under mild operating conditions. These biocatalysts allow fine control over key process variables—such as enzyme loading, temperature, pH, and reaction time—facilitating the adaptation of hydrolysis strategies to distinct lignocellulosic feedstocks. Table 4 shows studies reporting hemicellulose enzymes for degradation of lignocellulosic biomass.

**TABLE 3:** Factors that influence the structural features on xylooligosaccharides production.

Variable	Effect
GH10 enzymes	Lower degree of polymerization
GH11 enzymes	Higher degree of polymerization
pH	Cleavage pattern
Temperature	Hydrolysis rate
Reaction time	Degree of polymerization
Accessory enzymes	Side chain removal
Pretreatment severity	Xylan accessibility

**TABLE 4:** Fungi production of hemicellulose enzymes for degradation of lignocellulosic biomass.

Microorganism	Enzymes produced	Family	Substrate/inducer	Optimum pH	Optimum temperature (°C)	Reported activity and application	Reference
*Trichoderma harzianum* M7	Endo‐*β*‐1,4‐xylanase (SSF)	GH10, GH11	Sugarcane bagasse, water hyacinth	5.0–6.0	50–55	High xylanase yield (1724 U/g DM), suitable for biomass hydrolysis	(Losada‐Garcia et al. 2025)
*Aspergillus austwickii* B6	Xylanase (SSF)	GH11	Water hyacinth biomass	5.5	50	Efficient hemicellulose degradation using agro‐residues	(Losada‐Garcia et al. 2025)
*Talaromyces amestolkiae*	Endoxylanase, *β*‐xylosidase (SmF)	GH10, GH43	Beechwood xylan	4.5–5.5	50	Selective XOS production with low xylose formation	(Méndez‐Líter et al. 2021)
*Penicillium citrinum*	Xylanase (SSF)	GH11	Pretreated date seed powder	5.0	50	High xylanase activity (≈600 U/g), effective hemicellulose hydrolysis	(Alodaini and Hatamleh 2025)
*Myceliophthora heterothallica*	Endo‐*β*‐1,4‐xylanase (SmF)	GH10	Wheat bran, beechwood xylan	5.0–6.0	40–50	Thermotolerant enzyme suitable for lignocellulose bioconversion	(de Oliveira Simões et al. 2019)

Moreover, the integration of optimized pretreatment methods with tailored enzymatic systems has emerged as a critical approach to overcoming biomass heterogeneity while preserving xylan backbone integrity. Despite these advances, further progress is required to improve downstream fractionation efficiency and reduce enzyme‐related costs at scale. Importantly, aligning bioprocess design with functional outcomes remains a priority, reinforcing the need for multidisciplinary strategies that connect XOS structural features with microbiome modulation and host metabolic responses (Dong et al. 2023; Qin et al. 2022).

Despite the overall trend toward improved XOS yields and process efficiencies reported in recent studies, substantial variability remains across different experimental systems. Table 5 presents studies with enzymatic production of XOS obtained from lignocellulosic biomass. For instance, while several reports indicate high conversion efficiencies using commercial endo‐xylanases under optimized conditions, other studies employing similar substrates and enzymes have achieved considerably lower yields. These discrepancies may arise from differences in biomass pretreatment severity, enzyme purity, reaction time, and substrate accessibility (Balthazar et al. 2022). Furthermore, variations in lignin content and hemicellulose structure among feedstocks can significantly influence hydrolysis performance, limiting direct comparability between studies. Consequently, reported efficiencies should be interpreted cautiously, particularly in the absence of standardized protocols.

**TABLE 5:** Enzymatic production of xylooligosaccharides (XOS) from lignocellulosic biomass using predominantly fungal‐derived enzymes.

Biomass feedstock	Pretreatment	Enzyme source	Enzyme loading	Hydrolysis conditions	XOS yield and distribution	Reference
Corncob	Alkaline (NaOH)	Thermostable endo‐xylanase (recombinant, *Pichia pastoris*)	10–20 U g^−^^1^ xylan	55°C–60°C; pH 5.5; 12–24 h	∼65% XOS; predominantly X2 (≈45%–50%), X3 (≈25%–30%), minor X4–X5	(Shi et al. 2025)
Dissolving pulp residual hemicellulose	None (isolated hemicellulose)	Commercial endo‐xylanase	5–10 U g^−^^1^ substrate	50°C; pH 5.0; 6–12 h	∼43% XOS; X2 and X3 accounted for > 70% of products	(Wang et al. 2018)
Oil palm frond xylan	Alkaline extraction	Commercial fungal xylanase cocktail	10–30 U g^−^^1^ xylan	50°C–55°C; pH 5.0; 24 h	60%–80% XOS; X2–X4 dominant, limited monomer release	(Ahmad Sobri et al. 2023)
Poplar wood	Acetic acid + HPAC	Xylanase‐rich enzyme cocktail	∼15 FPU g^−^^1^ biomass	50°C; pH 4.8; 48 h	∼6.9 g XOS/100 g biomass; X2–X3 predominant	(Wen et al. 2019)
Wheat bran xylan	Extraction	Xylanase (*Penicillium rubens*)	5–15 U g^−^^1^ xylan	45°C–50°C; pH 5.0; 16 h	High selectivity toward X2 (xylobiose) and X3	(Šekuljica et al. 2023)
Eucalyptus hemicellulose	Extraction	Commercial xylanase + accessory enzymes	10–20 U g^−^^1^ xylan	50°C; pH 5.0; 24 h	∼30% conversion; X2–X4 major products	(Mafei and Masarin 2017)
Corncob, wheat bran	Alkaline extraction	Crude thermostable xylanase (*Scytalidium thermophilum*)	∼10 U g^−^^1^ xylan	55°C–60°C; pH 5.5; 24 h	Predominantly X2–X3; minimal xylose	(Kocabas and Ozben 2014)
Sugarcane bagasse xylan	Alkaline	Endo‐*β*‐1,4‐xylanase (low *β*‐xylosidase) *Aspergillus fumigatus* M51	∼15 U g^−^^1^ xylan	50°C; pH 5.0; 24 h	∼37% XOS; enriched in X2 and X3	(Azevedo Carvalho et al. 2015)
Wheat bran	None	Crude extract of wild isolates (*Penicillium* spp.)	Not reported	45°C–50°C; pH 5.0	XOS enriched in X2–X3	(Teixeira et al. 2023)

## 6 XOS as Prebiotics

**FIGURE 4:**
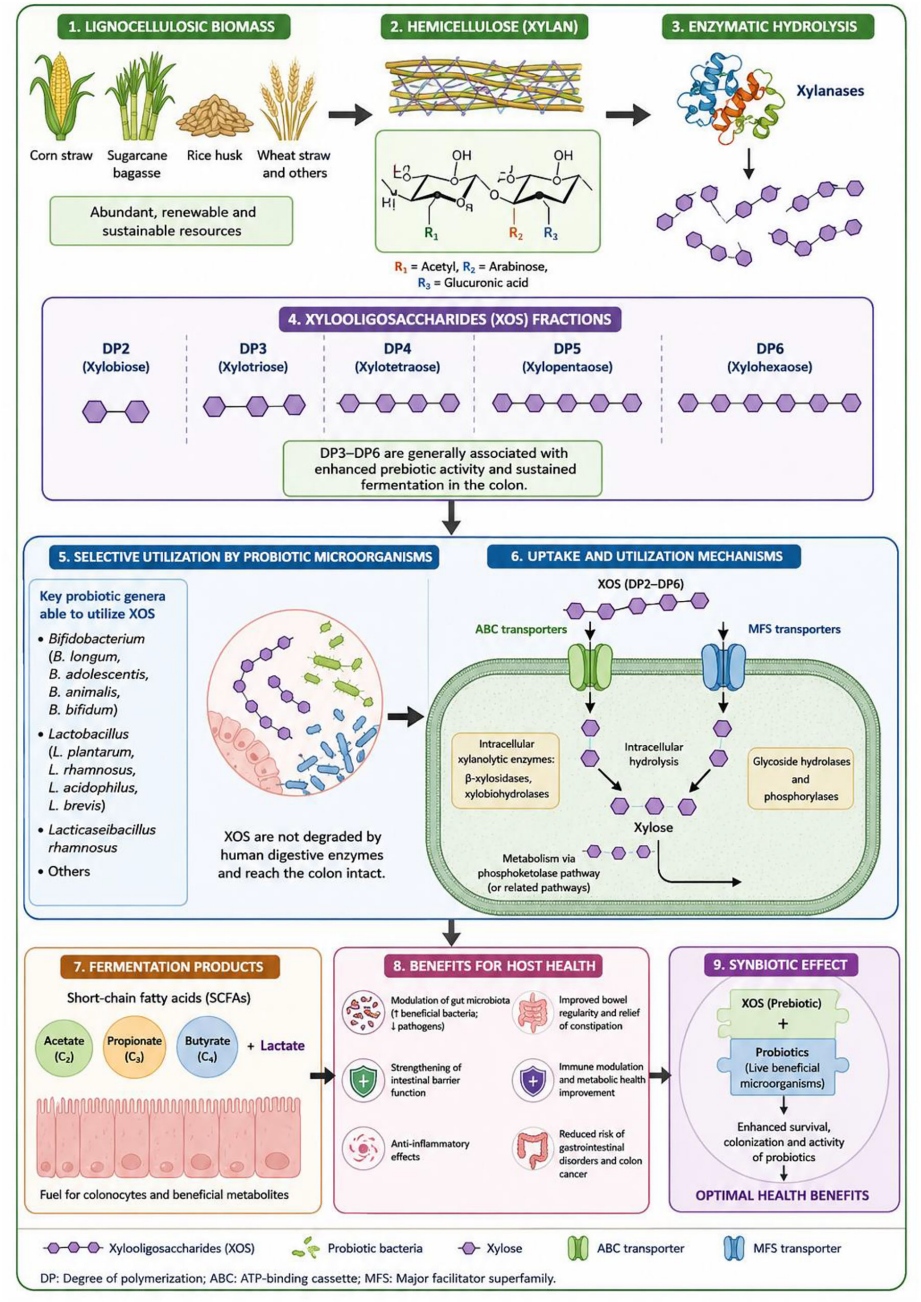
Metabolic health benefits associated with xylooligosaccharides consumption.

XOS are nondigestible oligosaccharides (NDOs) that are neither hydrolyzed nor absorbed in the upper gastrointestinal tract, which underlies their classification as prebiotics. Due to these properties, XOS selectively stimulate the growth and metabolic activity of beneficial gut microorganisms, particularly *Bifidobacterium* and *Lactobacillus* spp. (Chapla et al. 2012; Farias et al. 2019; Mohanty et al. 2018). Because human digestive enzymes are unable to cleave the β‐1,4‐glycosidic linkages of XOS, these oligosaccharides reach the lower gastrointestinal tract intact, where they are fermented by resident microbiota. During microbial fermentation, XOS are converted into SCFAs, such as acetate, propionate, and butyrate, which contribute to a reduction in luminal pH, inhibition of pathogenic microorganisms, and maintenance of intestinal barrier integrity (Zhao et al. 2021). These microbial and metabolic effects support a range of beneficial outcomes associated with XOS consumption, as summarized in Figure 4.

The functional properties of XOS are governed by two interrelated structural features: (i) DP, typically ranging from xylobiose (X2) to xylo‐oligomers of 6–7 units (X6–X7) in most functional preparations and (ii) the pattern and nature of side‐chain substitutions. Both DP and substitution pattern determine microbial selectivity, fermentability kinetics, and the types of SCFAs produced during fermentation. Consequently, production methods that allow controlled DP and preservation (or deliberate modification) of substitutions are essential when the aim is to produce XOS with predictable prebiotic activity (Finegold et al. 2014; Valladares‐Diestra et al. 2023).

There are several biological benefits of using XOS. They are currently used in the food, pharmaceutical, health, and cosmetic industries. The main interest in these five‐carbon sugars is due to their prebiotic potential, which positively affects human health. The XOS with the greatest prebiotic capacity are those with a DP between 2 and 6 (Amorim et al. 2019). In order to be regarded as prebiotics, the functional ingredient must follow a set of criteria defined below (Dong et al. 2023):

- Be nondigestible by the endogenous enzymes in the human gut. It should possess resistance toward gastric acidity, hydrolysis by digestive enzymes, and gastrointestinal tract absorption.
- Stimulate the growth and/or activity of specific bacteria responsible for health and wellbeing, thus, conferring health benefits to the host.
- Undergo selective fermentation by specific intestinal microbiota.
- Have been synthesized by a microorganism or their enzymes, or have plant origin.
- Be compatible with other food/feed ingredients.

**FIGURE 5:**
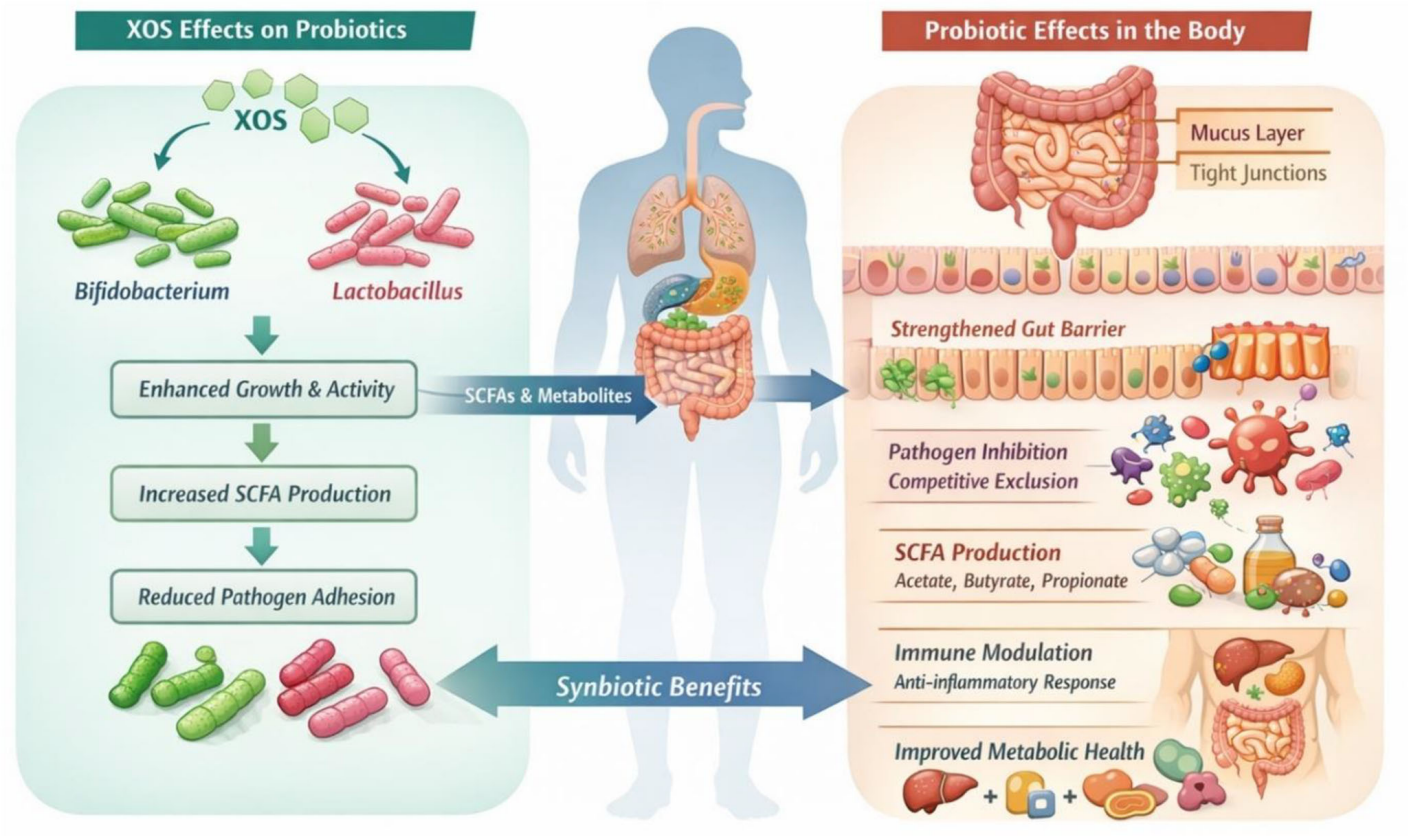
Xylooligosaccharide–probiotic interactions and their implications for gut health.

Prebiotics promote the modulation of the intestinal microbiota, increasing the production of SCFAs and reducing the risk of several diseases, such as IBS, colon cancer, obesity, among others (Farias et al. 2019). Figure 5 summarizes the potential interactions between XOS and probiotic microorganisms and their subsequent effects on host health. XOS can selectively stimulate the growth and activity of beneficial bacteria, promoting SCFA production and other microbial metabolites that may contribute to intestinal barrier integrity, pathogen inhibition, immune modulation, and metabolic health. Table 5 summarizes some recent studies in which XOS have been administered as prebiotics and their effects on different metabolic parameters.

The metabolic benefits associated with XOS administration are primarily mediated through selective modulation of the gut microbiota and its downstream metabolic activity. Due to their resistance to host digestive enzymes, XOS reach the colon intact, where they are preferentially utilized by saccharolytic bacteria, particularly *Bifidobacterium* spp. and other SCFA–producing taxa (Mäkeläinen et al. 2010; Yang et al. 2015; Zang et al. 2026). The enrichment of these microbial populations enhances the production of acetate, propionate, and butyrate, which act as key signaling metabolites linking microbial fermentation to host physiology (Fei et al. 2020). SCFAs activate G‐protein–coupled receptors (GPR41 and GPR43) on intestinal epithelial and immune cells, promoting improved glucose homeostasis, lipid metabolism, and anti‐inflammatory responses, while butyrate additionally serves as a histone deacetylase (HDAC) inhibitor, contributing to epithelial integrity and immune regulation (Chen et al. 2021; Li et al. 2021; Valladares‐Diestra et al. 2023) (Table 6).

**TABLE 6:** Recent reported studies on xylooligosaccharide (XOS) administration: doses, metabolic endpoints, and outcomes.

Study	Model	XOS dose	Key outcomes	Metabolic endpoint	Reference
Randomized human trial on gut microbiota	In vivo (human)	1.4 or 2.8 g/day for 8 weeks	Increased *Bifidobacterium* counts (dose‐dependent); no GI side effects	Microbiota modulation; no significant change in SCFA, pH	(Finegold et al. 2014)
Human prediabetic and healthy adults	In vivo (human)	2 g/day for 8 weeks	Reversed gut microbiota dysbiosis markers in pre‐DM; trend to reduce 2‐h insulin	Microbiota composition shifts; trend for improved glucose tolerance	(Yang et al. 2015)
High‐fat diet obese rats	In vivo (rat)	XOS (dose not specified) + HFD for 8–12 weeks	Alleviated weight gain; reduced plasma TNF‐α and MCP‐1; increased SCFA producers	↑ SCFA (acetate, propionate, butyrate); ↓ inflammation; ↑ occludin expression	(Lensu et al. 2020)
XOS in high‐fat diet mice	In vivo (mouse)	250 and 500 mg/kg for 12 weeks	Inhibited weight gain; improved lipid profile; enhanced gut barrier; optimized microbiota	↓ TG, TC, LDL‐C; ↑ ZO‐1, occludin, tight junction proteins	(Li et al. 2021)
XOS + high‐fat diet microbiota study	In vivo (mouse)	XOS included in HF diet	↑ Acetic, propionic, butyric acid; ↑ Bifidobacteria and Lachnospiraceae; tendency to ↓ weight gain	↑ SCFA, microbiota modulation	(Berger et al. 2021)
Stress‐induced behavior in vivo	In vivo (mouse)	0.23 g/kg‐day for 4 weeks	↑ Fecal SCFAs (acetate, propionate, butyrate)	Enhanced SCFA production; potential gut–brain axis effects	(Shu et al. 2022)
XOS + probiotic on glucose metabolism	In vivo (mouse, diabetic)	250 or 500 mg/kg XOS + *L. rhamnosus*	Low XOS dose with probiotic improved glycemia; high dose increased IR and inflammation	Dose‐dependent glucose metabolism effects	(Cui et al. 2023)
Corn cob XOS in constipation model	In vivo (mouse)	XOS (composition specific)	↑ GI transit, ↓ colonic inflammation	↑ SCFA, improved GI motility	(Song et al. 2023)

Despite the generally positive effects of XOS supplementation reported in both animal and human studies, notable inconsistencies remain across experimental models and intervention protocols. While several investigations demonstrate improvements in gut microbiota composition, inflammatory status, and metabolic markers, others report limited or negligible effects on SCFA production, glucose homeostasis, or lipid metabolism (Li et al. 2021). For instance, some human trials indicate dose‐dependent bifidogenic responses without significant changes in SCFA levels, whereas animal studies frequently report pronounced metabolic benefits under high‐fat diet conditions (Berger et al. 2021; Fei et al. 2020; Lensu et al. 2020). Furthermore, evidence of adverse effects at higher doses, including increased insulin resistance and inflammatory markers, highlights the nonlinear and context‐dependent nature of XOS–host interactions. These divergent outcomes suggest that microbial background, dietary composition, intervention duration, and XOS structural characteristics may critically modulate biological responses, thereby limiting direct comparability among studies (Ma et al. 2025; Zang et al. 2026).

Consistently, animal studies have demonstrated that XOS supplementation reduces circulating triglycerides and cholesterol, improves insulin sensitivity, and attenuates hepatic steatosis under high‐fat diet conditions, effects closely associated with increased SCFA availability and restoration of tight junction protein expression (Chen et al. 2021; Fei et al. 2020; Lensu et al. 2020; Li et al. 2021). Importantly, recent evidence indicates that these effects are dose‐dependent, as excessive XOS intake may exacerbate inflammatory signaling and impair glucose regulation when microbial fermentation exceeds host absorptive and regulatory capacity (Cui et al. 2023). In humans, lower daily doses (1.4–2.8 g/day) appear sufficient to induce bifidogenic effects without adverse gastrointestinal or metabolic outcomes, highlighting the importance of dosage optimization for translational applications (Ali et al. 2025; Carvalho et al. 2013; Corim Marim and Gabardo 2021; Finegold et al. 2014; Guarino et al. 2020; Jain et al. 2015; Sabanci et al. 2025). Collectively, these findings support a mechanistic framework in which XOS act as functional metabolic modulators by reshaping gut microbial ecology, enhancing SCFA‐mediated host signaling, and improving systemic metabolic homeostasis.

A major limitation underlying the current body of evidence relates to the substantial heterogeneity in experimental design, dosing strategies, and analytical methodologies. Many studies employ different XOS preparations with variable degrees of polymerization and substitution patterns, which are often insufficiently characterized. In addition, inconsistencies in dosage reporting, intervention periods, and dietary controls complicate the interpretation of dose–response relationships. The frequent reliance on small sample sizes, short‐term interventions, and simplified animal models further restricts the extrapolation of findings to human populations. Moreover, incomplete reporting of fermentation conditions, microbiota analysis pipelines, and statistical validation procedures compromises reproducibility and limits cross‐study integration. Standardized protocols and harmonized reporting frameworks are therefore essential to enhance the robustness and translational relevance of future research.

Potential sources of experimental and interpretative bias must also be considered when evaluating the reported functional effects of XOS. A substantial proportion of available evidence derives from in vivo animal models, which, although mechanistically informative, do not fully capture the complexity and interindividual variability of the human gut microbiome. In clinical studies, limited cohort diversity, short follow‐up periods, and insufficient control of confounding dietary and lifestyle factors may introduce systematic bias. Additionally, the predominance of studies reporting positive outcomes suggests the possible presence of publication bias, with negative or inconclusive results being underrepresented. Selective outcome reporting and limited transparency in data availability further constrain independent validation. Collectively, these biases may overestimate the efficacy of XOS and underscore the need for rigorously designed, long‐term, and adequately powered clinical trials.

## 7 Probiotics

Probiotics, classically defined as live microorganisms that confer health benefits to the host when administered in adequate amounts (FAO and WHO 2002), have evolved beyond traditional lactic acid bacteria to include next‐generation probiotics (NGPs) and spore‐forming strains with enhanced functionality and resilience. Well‐established probiotic genera such as *Lactobacillus* and *Bifidobacterium* remain central to clinical and industrial applications, exhibiting mechanisms of action that include competitive exclusion of pathogens, modulation of host immune responses, and production of metabolites such as SCFAs that support gut homeostasis (Markowiak‐Kopeć and Śliżewska 2020).

In addition to these traditional strains, spore‐forming bacteria such as *Bacillus coagulans* have gained prominence due to their exceptional stability under processing and gastrointestinal conditions, resisting heat, desiccation, and acid challenges that often impair the viability of non‐spore probiotics (Cutting 2011; Hong et al. 2005; Konuray and Erginkaya 2018; Majeed and Kamarei 2012). Such robustness makes *B. coagulans* particularly attractive for incorporation into functional foods, beverages, and supplements that undergo thermally intensive processing.

More recently, the concept of NGPs has emerged, encompassing microorganisms with promising health effects beyond traditional species, often isolated from the human gut microbiome and with unique metabolic capabilities. Examples include *Akkermansia muciniphila*, a mucin‐degrading bacterium associated with improved metabolic health and reduced inflammation in preclinical and early clinical studies; *Faecalibacterium prausnitzii*, known for its anti‐inflammatory properties through butyrate production; and certain strains of *Roseburia* and *Eubacterium* that contribute to butyrate pools and gut barrier integrity (Cani and de Vos 2017; Pryde et al. 2002; Zhang et al. 2014). These NGPs are under active investigation for applications in metabolic disorders, inflammatory bowel disease, and even neuroimmune modulation.

Cutting‐edge research has also explored the use of engineered probiotic strains that express therapeutic molecules or enhance native metabolic pathways. For instance, genetically modified *Lactococcus lactis* strains have been developed to deliver anti‐inflammatory cytokines in models of colitis, while commensal *Escherichia coli* Nissle derivatives are being tested as platforms for modulating gut metabolite production (Isabella et al. 2018; Mimee et al. 2016).

According to Food and Agriculture Organization (FAO)/World Health Organization (WHO), microorganisms with probiotic potential, in order to guarantee their viability and functionality, must meet the following criteria (FAO and WHO 2002):

- do not show genetic variation;
- be stable in the food over its shelf life and be resistant to the shelf life;
- be resistant to the acid environment of the stomach and salts bile;
- have the ability to proliferate and survive in the intestine;
- produce metabolites;
- modulate the immune system;
- belong to the Food and Drug Administration (FDA) list of generally regarded as safe (GRAS), with the possibility of resisting antibiotics.

Probiotics are isolated from the gut and fermented foods. They can be considered as GRAS according to the US FDA, or as qualified presumption of safety (QPS) according to the European Food Safety Authority (EFSA) (Martín and Langella 2019).

It is known that over 1000 microbial species inhabit the human gastrointestinal tract, which constitutes a complex community of microorganisms entitled gut or intestinal microbiota. Approximately 10^14^ microorganisms compose this ecosystem, most of them from the group of anaerobic bacteria. Alterations in this complex ecological system have demonstrated correlation to diseases, such as IBS, encompassing ulcerative colitis, Crohn's disease, Type 2 diabetes, and obesity (Guinane and Cotter 2013; Linares‐Pasten et al. 2016; Liu et al. 2024).

Studies carried out using 16S ribosomal RNA analysis showed three phyla of beneficial individuals have been identified as having the greatest quantity: *Firmicutes*, *Bacteroidetes*, and *Actinobacteria*, including the genera most reported in the literature with this beneficial effect: *Bifidobacterium* spp. and *Lactobacillus* spp. This intestinal microbiota varies in each person, at least at the species level. The main functions of the intestinal microbiota are the formation of the intestinal wall, resistance against pathogens, production of vitamins (mainly B and K complex), interactions with the immune system, improving the availability of nutrients and nondigestible compounds, among others (Martinez et al. 2015).

One critical pathway by which probiotics exert systemic effects is through the fermentation of dietary substrates and the consequent production of SCFAs, such as acetate, propionate, and butyrate (Chandrasekaran et al. 2024; Markowiak‐Kopeć and Śliżewska 2020). SCFAs are not only a key energy source for colonocytes, but also act as signaling molecules: They bind to G‐protein‐coupled receptors (e.g., GPR41, GPR43) on host cells, modulating immune function, strengthening epithelial barrier integrity, and reducing inflammation (Markowiak‐Kopeć and Śliżewska 2020).

Probiotics can promote anti‐inflammatory cytokine production, support regulatory T cell populations, and help maintain immune homeostasis through interactions with immune cells, such as dendritic cells and T lymphocytes. Moreover, certain strains can produce neuroactive compounds (e.g., γ‐aminobutyric acid, serotonin, dopamine), which may contribute to gut–brain axis signaling, impacting mood, stress, and gut motility (Latif et al. 2023).

Probiotics’ mechanism of action are multifactorial, including competitive exclusion of pathogens by occupying binding sites and competing for nutrients, enhancement of the intestinal barrier via upregulation of tight junction proteins and mucin production, and immunomodulation of both innate and adaptive immune responses (Latif et al. 2023; Markowiak‐Kopeć and Śliżewska 2020).

They also have primary mechanisms for inhibiting the development of pathogenic microorganisms: pH reduction through the production of lactic acid; production of antimicrobial substances, such as bacteriocin, hydrogen peroxide, and compounds such as free radicals; competition for food resources, also called competitive exclusion of microorganisms; stimulation of IgA (immunoglobulin A) production, through the formation of protective mucin, which is a mucus‐like substance composed of tissue of epithelial or conjunctival origin, and a mixture of glycoproteins with mucus proteins; and through modulation of the immune system (Bermudez‐Brito et al. 2012; Konuray and Erginkaya 2018).

Despite the extensive body of literature supporting the health‐promoting effects of probiotics, considerable heterogeneity persists in reported outcomes across clinical and preclinical studies. While traditional genera such as *Lactobacillus* and *Bifidobacterium* consistently demonstrate beneficial effects on gut homeostasis and immune modulation, evidence regarding NGP and engineered strains remains largely preliminary and context‐dependent. Many reported benefits are derived from animal models or small‐scale human trials, limiting their generlizability. Moreover, strain‐specific differences, variable colonization capacity, and strong dependence on host microbiome composition contribute to inconsistent therapeutic responses. As a result, extrapolation of findings across different populations, formulations, and health conditions remains challenging, underscoring the need for large, well‐controlled, multicenter clinical studies (Cani and de Vos 2017; Hua et al. 2025; Jadhav et al. 2026; Nhung et al. 2025).

Methodological limitations further constrain the interpretation and translation of probiotic research. A lack of standardization in strain identification, dosing regimens, formulation stability, and outcome assessment hampers reproducibility and cross‐study comparison. In addition, many studies rely on short intervention periods and surrogate biomarkers, which may not adequately reflect long‐term health effects. The predominance of positive findings suggests the possible influence of publication and sponsorship bias, particularly in commercially driven research.

Furthermore, regulatory classifications such as GRAS and QPS primarily address safety rather than efficacy, potentially creating a gap between regulatory approval and clinically validated benefit. Collectively, these factors highlight the urgent need for harmonized methodological frameworks, transparent reporting practices, and rigorous post‐market evaluation to ensure that probiotic‐based interventions are both scientifically robust and clinically meaningful (Kim et al. 2026).

## 8 Symbiotic Interactions Between Prebiotic XOS and Probiotic Microorganisms

XOS, generated through controlled enzymatic hydrolysis of hemicellulosic fractions of LCB, function as selective prebiotics by resisting hydrolysis in the upper gastrointestinal tract and reaching the colon intact, where they exert their prebiotic effects primarily through selective utilization by beneficial gut microorganisms, particularly *Bifidobacterium* and *Lactobacillus* species (Aachary and Prapulla 2011; Gibson et al. 2004, 2017). This selective fermentation stimulates probiotic growth and metabolic activity, leading to increased production of SCFAs, such as acetate, propionate, and butyrate (Markowiak‐Kopeć and Śliżewska 2020). These microbial metabolites play a central role in host health by strengthening intestinal barrier integrity, modulating immune responses, suppressing pathogenic microorganisms, and contributing to systemic metabolic regulation (Lebeer et al. 2008; Sanders et al. 2019). In parallel, probiotics interact directly with intestinal epithelial and immune cells, influencing inflammatory signaling pathways and promoting gut microbiota homeostasis. The synergistic relationship between XOS and probiotics therefore underpins symbiotic functionality, establishing a mechanistic link between sustainable lignocellulosic bioconversion processes and gut microbiota modulation with consequent host health benefits (Gibson et al. 2017).

Recent advances in gut microbiome research have demonstrated that the prebiotic activity of XOS is highly dependent on the metabolic capabilities of specific probiotic microorganisms (Aachary and Prapulla 2011; Finegold et al. 2014; Moura et al. 2007). Among the most extensively studied XOS‐utilizing bacteria are *Bifidobacterium adolescentis, Bifidobacterium longum, Bifidobacterium animalis, Lactobacillus plantarum, Lactobacillus rhamnosus*, and *Lactobacillus acidophilus* (Falck et al. 2013; Rivière et al. 2016). These microorganisms possess specialized carbohydrate utilization systems that enable the uptake and metabolism of xylose‐based oligosaccharides, thereby contributing to the selective nature of XOS fermentation and supporting their application in synbiotic formulations (Markowiak‐Kopeć and Śliżewska 2020; Patel and Goyal 2012) (Table 7).

**TABLE 7:** Synbiotic effects of xylooligosaccharides (XOS) combined with probiotics: evidence from in vitro, animal, and human studies.

Study	XOS source/dose	Probiotics used	Duration	Key findings	Reference
Human dietary intervention	2 g/day XOS	*Lactobacillus rhamnosus* GG	8 weeks	↑ *Bifidobacterium* and *Lactobacillus* counts; ↓ *Clostridium perfringens*; improved markers of gut health	(Lin et al. 2016)
Rodent metabolic syndrome model	500 mg/kg XOS	*Lactobacillus rhamnosus* + *Bifidobacterium longum*	8 weeks	↓ Serum TG, TC, and LDL; ↑ SCFA (acetate, butyrate); improved glucose tolerance	(Cui et al. 2023)
In vitro fecal fermentation	3% XOS (corn fiber)	*Bifidobacterium adolescentis*	48 h	Selective stimulation of *Bifidobacterium* growth; ↑ acetate and propionate; reduced pH	(Amorim et al. 2020)
Mouse colitis model	250 mg/kg XOS	*Lactobacillus acidophilus* + *Bifidobacterium bifidum*	6 weeks	Attenuated colonic inflammation; ↑ gut barrier proteins (occludin); ↑ SCFA; ↑ beneficial taxa	(Deng et al. 2023)
In vitro intestinal barrier model	1% (w/v)	*Bifidobacterium pseudocatenulatum* E‐0810	48 h	↑ SCFA production; suppression of NF‐κB signaling; ↑ epithelial barrier integrity	(Yang et al. 2025)

Members of the genus *Bifidobacterium* are considered the primary consumers of XOS in the human gut (Falck et al. 2013; Rivière et al. 2016). Genomic and transcriptomic studies have identified dedicated ATP‐binding cassette (ABC) transporters responsible for the efficient uptake of XOS molecules into the bacterial cell (Lagaert et al. 2014). Once internalized, intracellular β‐xylosidases and other GHs catalyze the hydrolysis of XOS into xylose monomers, which subsequently enter the bifid shunt pathway for energy generation and biomass production (Pokusaeva et al. 2011; Rivière et al. 2016). *Bifidobacterium adolescentis* and *Bifidobacterium longum* exhibit particularly high affinity toward short‐chain XOS fractions (DP2–DP5), resulting in pronounced bifidogenic effects and increased acetate production (Falck et al. 2013; Finegold et al. 2014; Moura et al. 2007). The interaction between XOS and bifidobacteria represents a classical synbiotic mechanism, in which the prebiotic selectively stimulates the growth and metabolic activity of probiotic microorganisms, enhancing their persistence and functionality within the gastrointestinal tract (Gibson et al. 2004; Markowiak‐Kopeć and Śliżewska 2020).

*Lactobacillus* species also participate in XOS metabolism, although generally with lower efficiency than bifidobacteria (Aachary and Prapulla 2011; Patel and Goyal 2012). Strains such as *Lactobacillus plantarum, Lactobacillus rhamnosu*s, and *Lactobacillus acidophilus* possess carbohydrate transport systems and intracellular xylosidases that enable partial utilization of XOS (Jenkins et al. 2010; Patel and Goyal 2012). Their growth on XOS contributes to enhanced lactic acid production and supports intestinal barrier integrity, immune modulation, and pathogen exclusion (Markowiak‐Kopeć and Śliżewska 2020; Sanders et al. 2019). The ability of these microorganisms to metabolize XOS varies considerably among strains, reflecting differences in carbohydrate‐active enzyme repertoires and regulatory mechanisms (Lagaert et al. 2014; Rivière et al. 2016). In synbiotic systems, XOS can improve probiotic survival during gastrointestinal transit, promote adhesion to intestinal epithelial cells, and enhance colonization efficiency, thereby strengthening probiotic‐mediated health benefits (Markowiak‐Kopeć and Śliżewska 2020; Patel and Goyal 2012).

An important feature of XOS fermentation is the establishment of cross‐feeding interactions within the gut microbiota (Louis and Flint 2017; Rivière et al. 2016). During primary fermentation, bifidobacteria convert XOS into metabolites such as acetate and lactate through the fructose‐6‐phosphate phosphoketolase pathway (Pokusaeva et al. 2011; Rivière et al. 2016). These compounds subsequently serve as substrates for secondary fermenters, particularly *Faecalibacterium prausnitzii* and other butyrate‐producing bacteria (Louis and Flint 2017; Pryde et al. 2002). This metabolic cooperation results in increased butyrate production, an SCFA associated with maintenance of intestinal epithelial integrity, anti‐inflammatory effects, and improved metabolic health (Koh et al. 2016; Louis and Flint 2017). Therefore, the health‐promoting effects of XOS extend beyond direct stimulation of probiotic bacteria and involve complex trophic networks within the gut microbial ecosystem (Gibson et al. 2017; Rivière et al. 2016).

The mechanisms underlying XOS–probiotic interactions can be summarized as follows: (i) selective utilization of XOS by probiotic bacteria through specific ABC transporters and GHs; (ii) stimulation of probiotic growth and metabolic activity, leading to increased production of acetate and lactate; (iii) enhancement of probiotic survival, colonization, and adhesion to the intestinal mucosa; (iv) competitive exclusion of pathogenic microorganisms through acidification of the intestinal environment and production of antimicrobial metabolites; (v) modulation of host immune responses via interactions between microbial metabolites and intestinal immune cells; and (vi) promotion of cross‐feeding interactions that increase butyrate production by commensal bacteria such as *Faecalibacterium prausnitzii* (Gibson et al. 2017; Louis and Flint 2017; Markowiak‐Kopeć and Śliżewska 2020; Sanders et al. 2019).

Collectively, these findings demonstrate that the synbiotic interaction between XOS and probiotic microorganisms is mediated by highly specialized carbohydrate transport and degradation systems, including ABC transporters and intracellular xylosidases, which ultimately drive SCFA production, beneficial microbiome modulation, enhanced probiotic functionality, and improved host health outcomes (Gibson et al. 2017; Markowiak and Śliżewska 2017; Rivière et al. 2016).

In addition to controlled in vitro and in vivo models, ex vivo systems have demonstrated that XOS are metabolized by colon microbiota across different intestinal sections, with significant SCFA production and selective stimulation of beneficial genera such as *Bifidobacterium* and *Lactobacillus*—mechanisms that support synergistic interactions in synbiotics formulations (Sabanci et al. 2025).

Despite significant progress in elucidating the production processes and functional properties of XOS, the current body of evidence remains fragmented, inconsistent, and characterized by substantial methodological heterogeneity. Conflicting results regarding production efficiency, structural optimization, and biological activity reflect variations in substrate composition, enzyme systems, and experimental design. Methodological limitations, including limited scalability, incomplete parameter reporting, and insufficient statistical validation, further restrict data comparability. Additionally, experimental and publication biases—particularly the overrepresentation of short‐term and small‐scale studies—may inflate perceived efficacy. Collectively, these challenges highlight the urgent need for harmonized protocols, multicenter validation studies, and integrative analytical frameworks to support robust translational progress (Kim et al. 2026; Meijerink et al. 2013).

Although the metabolic benefits associated with XOS and probiotic supplementation appear promising, it is important to recognize that a substantial proportion of the available evidence remains preclinical in nature. Many reported effects are derived from in vitro fermentation systems, animal models, or small‐scale human studies with limited statistical power and short intervention periods. While these approaches provide valuable mechanistic insights, they do not fully capture the complexity, interindividual variability, and long‐term dynamics of human metabolic regulation.

Collectively, these findings demonstrate that XOS are not merely prebiotic substrates but key components of synbiotic systems. Through selective utilization by probiotic microorganisms, stimulation of SCFA production, enhancement of probiotic survival, and promotion of cross‐feeding interactions within the gut microbiota, XOS establish a mechanistic bridge between LCB valorization and human health. Therefore, the development of tailored XOS fractions from renewable biomass sources represents a promising strategy for the formulation of next‐generation synbiotic products with applications in functional foods, nutraceuticals, and personalized nutrition.

## 9 Conclusion

This review highlights the central role of prebiotics and probiotics as key components of next‐generation functional foods, with particular emphasis on XOS as emerging, high‐value prebiotics derived from LCB. Advances in enzymatic bioconversion—especially those employing fungal hemicellulases—have enabled more selective and sustainable production of XOS with controlled DP (X2–X6), which are increasingly recognized as critical determinants of prebiotic efficacy. Compared with chemical hydrolysis routes, enzymatic approaches offer superior specificity, reduced formation of undesirable byproducts, and improved compatibility with food‐grade and environmentally responsible processes.

Accumulating in vitro, ex vivo, animal, and human studies consistently demonstrate that XOS selectively modulate gut microbiota composition, stimulate beneficial taxa such as *Bifidobacterium* and *Lactobacillus*, and enhance microbial metabolic outputs, particularly SCFAs. When combined with probiotic strains in synbiotic formulations, XOS further amplify these effects by promoting probiotic survival, colonization, and metabolic activity, ultimately translating into improvements in gut barrier function, immune modulation, and host metabolic health. These findings reinforce the concept that synbiotic efficacy arises from mechanistically linked interactions between substrate structure, microbial enzymatic capacity, and host responses, rather than from the independent actions of prebiotics or probiotics alone.

Despite these advances, several challenges remain before XOS‐based synbiotics can reach their full industrial and clinical potential. Variability in lignocellulosic feedstocks, heterogeneity in xylan structure, and differences in enzyme specificity complicate process standardization and scale‐up. Moreover, the economic feasibility of XOS production is tightly coupled to enzyme cost, reaction efficiency, and downstream purification strategies. From a biological standpoint, a major knowledge gap persists in systematically linking XOS structural features—such as DP and substitution patterns—to predictable microbiota shifts and host health outcomes in well‐designed human intervention studies.

Looking forward, the integration of biotechnological innovation with microbiome science will be essential to advance next‐generation synbiotics. This includes the rational design of enzyme cocktails, the use of precision fermentation, and valorization of agro‐industrial residues, and the selection of probiotic strains with defined glycan‐utilization pathways. Coupled with emerging regulatory frameworks and growing consumer demand for evidence‐based functional foods, these developments position XOS‐based synbiotics as promising tools for sustainable nutrition, gut health management, and disease risk reduction. Continued interdisciplinary research will be critical to translating mechanistic insights into robust, scalable, and clinically validated synbiotic products.

## Author Contributions

**Paula Cavion Costa**: conceptualization, writing – original draft, writing – review and editing, formal analysis, investigation, methodology, validation, data curation. **Marli Camassola**: conceptualization, writing – original draft, writing – review and editing, validation, formal analysis, project administration, resources, supervision, data curation, methodology, funding acquisition, investigation.

## Conflicts of Interest

The authors declare no conflicts of interest.
